# Infant Mortality and Maternal Risk Factors in Texas: Highlighting Zip Code Variations in 2 At-Risk Counties, 2011–2015

**DOI:** 10.5888/pcd19.210266

**Published:** 2022-01-13

**Authors:** Meliha Salahuddin, Krystin J. Matthews, Nagla Elerian, David L. Lakey, Divya A. Patel

**Affiliations:** 1 University of Texas Health Science Center, Tyler, Texas; 2 University of Texas System Office of Health Affairs, Austin, Texas; 3 University of Texas Health Science Center at Houston, School of Public Health, Austin, Texas

## Abstract

**Introduction:**

Stark differences in the infant mortality rate (IMR) exist by geography in Texas. The Healthy Families initiative sought to understand how evidence-informed practices implemented in the community can improve pregnancy-related outcomes in 2 counties in Texas with a high prevalence of maternal chronic conditions. The objective of this study was to examine associations between maternal risk factors and infant deaths to inform strategies to improve outcomes.

**Methods:**

Two counties with high prevalence of maternal chronic conditions were selected as Healthy Families sites: one with lower prenatal care usage than other counties in the state but an IMR lower than Texas, and the other with a higher IMR among minority racial and ethnic groups compared with other women in the county and Texas overall. Cohort-linked birth and infant death records from 2011 through 2015 provided by the Texas Department of State Health Services were analyzed by using logistic regression to examine associations of maternal sociodemographic and pregnancy risk factors with infant death. The data were mapped at the zip code level. Analyses were limited to births to women aged 15 to 49 years who resided in Texas from 2011 through 2015 (n = 1,942,899 births).

**Results:**

The Texas IMR was 5.4 per 1,000 live births, compared with 4.6 and 7.5 per 1,000 live births for Hidalgo and Smith counties, respectively. Congenital malformations were the leading cause of infant death in both counties for infants born in 2015, which was similar to Texas overall. In both counties, maternal marital status, education, multiple gestation, and cesarean delivery were significantly associated with infant mortality. Wide zip code–level variations in IMR and maternal risk factors were observed in both counties.

**Conclusion:**

Variations in IMR and key maternal risk factors observed at the zip code level helped drive local strategies to maximize outreach of services to disproportionately affected communities.

**Summary**

**What is already known on this topic?**

The infant mortality rate (IMR) in Texas is below the Healthy People 2020 objective; furthermore, stark differences in IMR exist within the state.

**What is added by this report?**

During 2011 through 2015 in 2 Texas counties, maternal sociodemographic and pregnancy-related characteristics were significantly associated with infant mortality. Wide zip code–level variations in the IMR and key maternal risk factors existed in both counties.

**What are the implications for public health practice?**

Findings from the study helped identify communities where potential scaling of effective interventions to improve pregnancy outcomes were needed and identify key strategies to address preconception and interconception health.

## Introduction

Although the infant mortality rate (IMR) in Texas has remained below the Healthy People 2020 objective of 6.0 per 1,000 live births (1) since 2012, wide variation in the IMR exists across zip code areas in the state, with some zip codes having as many as 20 deaths per 1,000 live births in 2011 through 2014 (2). Further, racial and ethnic minority disparities in the IMR persist in Texas, with the IMR being 2 times higher for non-Hispanic Black infants compared with that of non-Hispanic White or Hispanic infants (3).

The prevalence of chronic maternal health conditions, which are linked to poor pregnancy-related outcomes, is also increasing in Texas. Prepregnancy obesity, which leads to various complications during pregnancy, has increased about 30% in Texas since 2009, with Black and Hispanic women having the highest rates (3). Hypertension and diabetes are also increasing among mothers in Texas, with Black and Hispanic women having the highest rate for hypertension and diabetes, respectively (3).

Given the high prevalence of racial and ethnic minority disparities in infant mortality and associated maternal risk factors, there is growing urgency to move evidence-informed research to practice and policy. The Healthy Families initiative was launched in fall 2016 by the Texas Health and Human Services Commission (HHSC), the agency that administers Medicaid and other women’s health programs, with the overall goal of understanding multilevel contextual factors influencing pregnancy outcomes in populations that have low access to state-funded prenatal care and poor maternal and infant health outcomes (4). The 4-year initiative was a unique partnership between a state agency, academic institutions, and 2 communities. The HHSC provided flexible funding to support identification, development, implementation, adaptation, and evaluation of evidence-informed practices to address community-identified gaps in pregnancy outcomes (4). As part of the Healthy Families evaluation, secondary data analyses using vital records data were conducted to drive strategies to focus on evidence-informed practices in the disproportionately affected communities in the 2 counties. The goals of the study were to 1) identify individual-level factors influencing the IMR in the 2 selected counties in Texas that participated in the Healthy Families initiative; 2) identify the leading causes for infant deaths in the 2 counties in comparison to Texas; and 3) describe zip code–level variation in the IMR and associated key maternal risk factors in the 2 counties. Findings from these analyses were integrated into the Healthy Families initiative to inform the planning, adaptation, and implementation of evidence-informed programs and strategies to address the IMR in the 2 project sites.

## Methods

### Healthy Families study setting

Texas HHSC selected 2 Texas counties, Hidalgo and Smith, based on county-level maternal and infant health indicators, as project sites for the Healthy Families initiative. Hidalgo County is in South Texas along the US–Mexico border and has lower prenatal care usage than other counties in the state but an IMR lower than Texas. Smith County is southeast of Dallas and has a higher IMR among minority racial and ethnic groups compared with other women in the county and Texas overall. US Census data indicate that both counties have median household incomes below the state level (5). The percentage of the population living below federal poverty guidelines is 12.9% in Smith County and 26.9% in Hidalgo county. The framework for the Healthy Families initiative and additional details about the 2 project sites have been described previously (4).

We conducted a cross-sectional secondary data analysis of cohort-linked birth and infant death records for 2011 through 2015 provided by the Texas Department of State Health Services Center for Health Statistics, separately for Hidalgo County (n = 80,799) and for Smith County (n = 15,269), and for Texas overall (n = 1,989,757). We limited our analyses to women of reproductive age (15–49 y) who were Texas residents. For Texas overall, women who were not aged 15 to 49 years (n = 2,790) or who did not have an address in Texas (n = 44,068) were excluded. Analyses were limited to births to women aged 15 to 49 years who resided in Texas in 2011 through 2015 (n = 1,942,899 births). The research study was approved by the Texas Department of State Health Services Institutional Review Board (IRB#17–055).

### Measures

The outcome of interest was infant mortality, defined as death of an infant before his or her first birthday, and was operationalized dichotomously, from the Texas linked live birth–infant cohort files for 2011 through 2015. To protect confidentiality and obtain the most accurate estimates while accounting for small frequencies for infant deaths, the 2011 through 2015 files were aggregated.

The exposures of interest were maternal sociodemographic factors and pregnancy-related characteristics. Sociodemographic factors were maternal age, education (categorized as high school graduate or less and some college or more), marital status (categorized as currently married or not), maternal race and ethnicity (categorized as Hispanic, non-Hispanic Black, non-Hispanic White, and other or unknown [American Indian or Alaska Native, Asian Indian, Chinese, Filipino, Japanese, Korean, Vietnamese, other Asian, Native Hawaiian, Guamanian or Chamorro, Samoan, Other Pacific Islander, and other — not specified]), nativity (categorized as US born or not), and principal source of payment for health services (categorized as private insurance, Medicaid, and other or self-pay). Pregnancy-related characteristics were maternal cigarette smoking during pregnancy, maternal prepregnancy obesity, preexisting or gestational diabetes, prenatal care, preexisting or gestational hypertension or eclampsia, multiple gestation (pregnant with more than 1 fetus), mother transferred for maternal or fetal indicators for delivery, and final delivery route.

All the variables are collected on the standard birth certificate and fetal death report (6). Texas implemented the revised birth certificate in 2005 and the revised fetal death report in 2006 (6). On the Texas birth certificate, preexisting and gestational diabetes are mutually exclusive conditions, as are preexisting hypertension, gestational hypertension, and eclampsia. The indication for gestational hypertension includes pregnancy-induced hypertension and preeclampsia (6). Information on cigarette smoking during pregnancy is collected as an average number of cigarettes or packs of cigarettes smoked per day during the first, second, and third trimester of pregnancy. For the purposes of this analysis, cigarette smoking was categorized as a binary variable. Mother’s body mass index (BMI) was calculated based on her prepregnancy height and weight reported on the birth certificate (weight [in pounds] divided by height [in inches and squared] and the quotient multiplied by 703) (7). The Centers for Disease Control and Prevention (CDC) 2000 growth charts were used to calculate mother’s age-specific BMI percentile for those aged 15 to 19 years (8). Prepregnancy weight status classified as either underweight/normal/overweight or obese was created based on age-specific BMI percentile thresholds and for those aged 15 to 19 years (9) and BMI thresholds per CDC cut points for those aged 20 years or older (10). For descriptive purposes, prenatal care was classified as yes if the mother received any prenatal care and no if the mother did not receive any prenatal care. To capture more of the variability within prenatal care, prenatal care was also assessed based on the Adequacy of Prenatal Care Utilization Index and operationalized as inadequate prenatal care, defined as prenatal care that began after the fourth month of pregnancy with the mother having less than 50% of recommended prenatal care visits, versus other categories combined (intermediate to adequate plus, ie, prenatal care that began by the fourth month of pregnancy with the mother having 50% or more of recommended prenatal care visits) (11).

### Statistical analysis

Descriptive statistics were means (SDs) and frequencies and percentage depending on the type of variable. Bivariate analyses were conducted to examine differences in the variables by infant mortality, separately for the 2 counties and for Texas overall. Multiple logistic regression models were used to examine associations of maternal sociodemographic and pregnancy-related factors with infant death, separately for the 2 counties and for Texas overall. Less than 1% of the data for the exposures were missing for the 2 counties, and approximately 10% of the data for the exposures were missing for the overall Texas model. Thus, we conducted an available case analysis. The a priori significance was set at α = .05. Estimates are presented as adjusted odds ratios (aORs) with 95% CIs. In addition, we performed a sensitivity analysis by using an alternative approach of multiple imputation using chained equations to account for missing data that were assumed to be missing at random for the overall Texas model. Twenty-five data sets were imputed for the overall model that included the variables in the corresponding analytic model. We also compared sociodemographic characteristics of those with complete versus incomplete data for the overall Texas model. All analyses were conducted in SAS/STAT software (SAS Institute, Inc) and Stata 16.0 (StataCorp LLC).

The causes of infant deaths for Hidalgo County, Smith County, and Texas overall were based on the underlying cause of death and were determined following the procedures used by the National Center for Health Statistics to rank causes of deaths (12,13).

In addition, by using ArcGIS Desktop, version 10.4.1 (ESRI), we mapped the distribution of IMRs and county-specific key maternal risk factors (prepregnancy obesity, diabetes, hypertension, cigarette smoking during pregnancy, and prenatal care use) at the zip code level for Hidalgo and Smith counties. To obtain accurate data estimates and to control for small numbers, data for geographic areas with fewer than 100 births were suppressed. For zip code mapping purposes, we used the 2016 zip code boundaries from the ESRI Data and Maps (14).

## Results

### Maternal characteristics and infant mortality rate

The 2011 through 2015 IMRs in Hidalgo County and Smith County were 4.6 and 7.5 per 1,000 live births, respectively; the Texas IMR was 5.4 per 1,000 live births. In Hidalgo County, 97.0% of the women were Hispanic, 54.7% were married, and 35.1% had some college education, and they had a mean (SD) age of 26.4 (6.2) years. In Smith County, most women were either non-Hispanic White (49.8%) or Hispanic (29.5%), were married (57.0%), and had some college education (54.7%), and they had a mean (SD) age of 26.8 (5.7) years. Medicaid was the primary payment source for 46.6% of births in the state, 47.6% of births in Smith County, and 61.2% of births in Hidalgo County.

For Hidalgo County, a few factors differed significantly by infant death status: maternal education, maternal prepregnancy obesity, diabetes, multiple gestation, receipt of prenatal care, mother transferred for maternal or fetal indications, and delivery route (Table 1). For Smith County, factors that differed significantly by infant death status were marital status, maternal prepregnancy obesity, multiple gestation, receipt of prenatal care, mother transferred for maternal or fetal indications, and delivery route. However, for Texas overall, most factors differed by infant death status (Table 1).

**Table 1:** Maternal Sociodemographic and Pregnancy Characteristics, Overall and by Infant Death, Healthy Families Sites and Texas, 2011–2015^a^

Characteristic	Hidalgo County			Smith County			Texas		
	All Births (N = 80,621)	Infant Deaths		All Births (N = 15,253)	Infant Deaths		All Births (N = 1,942,899)	Infant Deaths	
		Yes, n = 368	No, n = 80,253		Yes, n = 115	No, n = 15,138		Yes, n = 10,622	No, n = 1,932,277
**Sociodemographic characteristics**									
Age, mean (SD), y	26.4 (6.2)	26.3 (6.9)	26.4 (6.2)	26.8 (5.7)	26.9 (5.8)	26.8 (5.7)	27.4 (6.0)	27.1 (6.5)^b^	27.4 (6.0)^b^
Missing	—	—	—	—	—	—	73	+	68
Education									
High school graduate or less	52,262 (64.8)	263 (71.5)^b^	51,999 (64.8)^b^	6,902 (45.3)	51 (44.4)	6,851 (45.3)	932,381 (48.0)	5,995 (56.4)^b^	926,386 (47.9)^b^
At least some college education	28,325 (35.1)	102 (27.7)^b^	28,223 (35.2)^b^	8,321 (54.7)	64 (55.7)	8,257 (54.5)	1,008,399 (52.0)	4,471 (42.1)^b^	1,003,928 (52.0)^b^
Missing	34 (0.0)	+	31 (0.0)	30 (0.2)	—	30 (0.2)	2,119 (0.1)	156 (1.5)	1,963 (0.1)
Marital status									
Married	44,090 (54.7)	198 (53.8)	43,892 (54.7)	8,699 (57.0)	52 (45.2)^b^	8,647 (57.1)^b^	1,126,048 (58.0)	5,261 (49.5)^b^	1,120,787 (58.0)^b^
Unmarried	36,531 (45.3)	170 (46.2)	36,361 (45.3)	6,554 (43.0)	63 (54.8)^b^	6,491 (42.9)^b^	816,829 (42.0)	5,361 (50.5)^b^	811,468 (42.0)^b^
Missing	—	—	—	—	—	—	22 (0.0)	—	22 (0.0)
Race and ethnicity									
Hispanic	78,216 (97.0)	359 (97.6)	77,857 (97.0)	4,493 (29.5)	28 (24.4)	4,465 (29.5)	928,453 (47.8)	4,687 (44.1)^b^	923,766 (47.8)^b^
Non-Hispanic Black	113 (0.1)	+	113 (0.1)	2,619 (17.2)	32 (27.8)	2,587 (17.1)	221,600 (11.4)	2,261 (21.3)^b^	219,339 (11.4)^b^
Non-Hispanic White	1,639 (2.0)	+	1,633 (2.0)	7,601 (49.8)	55 (47.8)	7,546 (49.9)	666,851 (34.3)	3,122 (29.4)^b^	663,729 (34.4)^b^
Other^c^ or unknown	621 (0.8)	+	618 (0.8)	519 (3.4)	—	519 (3.4)	123,304 (6.4)	495 (4.7)^b^	122,809 (6.4)^b^
Missing	32 (0.0)	—	32 (0.0)	21 (0.1)	—	21 (0.1)	2,691 (0.1)	57 (0.5)	2,634 (0.1)
US-born mother									
Yes	44,517 (55.2)	213 (57.9)	44,304 (55.2)	12,158 (79.7)	96 (83.5)	12,062 (79.7)	1,401,933 (72.2)	7,998 (75.3)^b^	1,393,935 (72.1)^b^
No	36,090 (44.8)	154 (41.9)	35,936 (44.8)	3,085 (20.2)	+	3,066 (20.3)	540,212 (27.8)	2,492 (23.5)^b^	537,720 (27.8)^b^
Missing	+	+	+	+	—	+	754 (0.0)	132 (1.2)	622 (0.0)
Principal source of payment									
Private insurance	11,465 (14.2)	48 (13.0)	11,417 (14.2)	6,219 (40.8)	49 (42.6)	6,170 (40.8)	732,167 (37.7)	3,207 (30.2)^b^	728,960 (37.7)^b^
Medicaid	49,359 (61.2)	237 (64.4)	49,122 (61.2)	7,261 (47.6)	58 (50.4)	7,203 (47.6)	905,873 (46.6)	5,471 (51.5)^b^	900,402 (46.6)^b^
Other or self-pay	19,758 (24.5)	82 (22.3)	19,676 (24.5)	1,754 (11.5)	+	1,747 (11.5)	302,080 (15.6)	1,906 (18.0)^b^	300,174 (15.5)^b^
Missing	39 (0.1)	+	38 (0.1)	+	+	+	2,779 (0.1)	38 (0.4)	2,741 (0.1)
**Pregnancy-related characteristics**									
Received prenatal care									
Yes	77,144 (95.7)	330 (89.7)^b^	76,814 (95.7)^b^	13,969 (91.6)	98 (85.2)^b^	13,871 (91.6)^b^	1,868,005 (96.2)	9,240 (87.0)^b^	1,858,765 (96.2)^b^
No	2,521 (3.1)	32 (8.7)^b^	2,489 (3.1)^b^	442 (2.9)	+	429 (2.8)^b^	57,882 (3.0)	1,049 (9.9)^b^	56,833 (3.0)^b^
Missing	959 (1.2)	+	950 (1.2)	842 (5.5)	+	838 (5.6)	17,012 (0.9)	333 (3.1)	16,679 (0.9)
Adequacy of Prenatal Care Utilization Index^d^									
Inadequate	10,578 (13.1)	40 (10.9)	10,538 (13.1)	3,198 (21.0)	20 (17.4)	3,178 (21.0)	328,303 (16.9)	1,624 (15.3)^b^	326,679 (16.9)^b^
Intermediate to adequate plus	44,113 (54.7)	197 (53.5)	43,916 (54.7)	10,521 (69.0)	73 (63.5)	10,448 (69.0)	1,429,091 (73.6)	6,607 (62.2)^b^	1,422,484 (73.6)^b^
Missing	25,930 (32.2)	131 (35.6)	25,799 (32.2)	1,534 (10.1)	22 (19.1)	1,512 (10.0)	185,505 (9.6)	2,391 (22.5)	183,114 (9.5)
**Presence of maternal risk factors**									
Any cigarette smoking during pregnancy									
Yes	226 (0.3)	^—^	226 (0.3)	1,054 (6.9)	+	1,042 (6.9)	81,112 (4.2)	749 (7.1)^b^	80,363 (4.2)^b^
No	80,391 (99.7)	366 (99.5)	80,025 (99.7)	14,194 (93.1)	103 (89.6)	14,091 (93.1)	1,861,588 (95.8)	9,858 (92.8)^b^	1,851,730 (95.8)^b^
Missing	+	+	+	+	+	+	199 (0.0)	+	184 (0.0)
Prepregnancy body mass index^e^									
Obesity	22,623 (28.1)	121 (32.9)^b^	22,502 (28.0)^b^	4,092 (26.8)	41 (35.7)^b^	4,051 (26.8)^b^	463,096 (23.8)	3,099 (29.2)^b^	459,997 (23.8)^b^
Overweight, normal, or underweight	57,842 (71.8)	243 (66.0)^b^	57,599 (71.8)^b^	11,083 (72.7)	72 (62.6)^b^	11,011 (72.8)^b^	1,468,790 (75.6)	7,248 (68.2)^b^	1,461,542 (75.6)^b^
Missing	156 (0.2)	+	152 (0.2)	78 (0.5)	+	76 (0.5)	11,013 (0.6)	275 (2.6)	10,738 (0.6)
Maternal diabetes^f^									
Yes	5,268 (6.5)	36 (9.8)^b^	5,232 (6.5)^b^	997 (6.5)	+	988 (6.5)	101,130 (5.2)	563 (5.3)	100,567 (5.2)
No	75,353 (93.5)	332 (90.2)^b^	75,021 (93.5)^b^	14,256 (93.5)	106 (92.2)	14,150 (93.5)	1,841,769 (94.8)	10,059 (94.7)	1,831,710 (94.8)
Missing	—	—	—	—	—	—	—	—	—
Maternal hypertension^f^									
Yes	4,564 (5.7)	25 (6.8)	4,539 (5.7)	1,213 (7.9)	+	1,201 (7.9)	129,940 (6.7)	913 (8.6)^b^	129,027 (6.7)^b^
No	76,057 (94.3)	343 (93.2)	75,714 (94.3)	14,040 (92.1)	103 (89.6)	13,937 (92.1)	1,812,959 (93.1)	9,709 (91.4)^b^	1,803,250 (93.3)^b^
Missing	—	—	—	—	—	—	—	—	—
Multiple gestation									
Yes	2,047 (2.5)	38 (10.3)^b^	2,009 (2.5)^b^	450 (3.0)	+	438 (2.9)^b^	62,768 (3.2)	1,406 (13.2)^b^	61,362 (3.2)^b^
No	78,574 (97.5)	330 (89.7)^b^	78,244 (97.5)^b^	14,803 (97.1)	103 (89.6)^b^	14,700 (97.1)^b^	1,880,119 (96.8)	9,216 (86.8)^b^	1,870,903 (96.8)^b^
Missing	—	—	—	—	—	—	+	—	+
Mother transferred for maternal or fetal indications for this delivery									
Yes	102 (0.1)	+	96 (0.1)^b^	104 (0.7)	+	101 (0.7)^b^	5,008 (0.3)	239 (2.3)^b^	4,769 (0.3)^b^
No	80,519 (99.9)	362 (98.4)^b^	80,157 (99.9)^b^	15,149 (99.3)	112 (97.4)^b^	15,037 (99.3)^b^	1,937,783 (99.7)	10,380 (97.7)^b^	1,927,403 (99.7)^b^
Missing	—	—	—	—	—	—	108 (0.0)	+	105 (0.0)
Final delivery route									
Vaginal	46,512 (57.7)	154 (41.9)^b^	46,358 (57.8)^b^	10,846 (71.1)	62 (53.9)^b^	10,784 (71.2)^b^	1,262,019 (65.0)	5,993 (56.4)^b^	1,256,026 (65.0)^b^
Cesarean	34,106 (42.3)	213 (57.9)^b^	33,893 (42.2)^b^	4,404 (28.9)	52 (45.2)^b^	4,352 (28.8)^b^	680,796 (35.0)	4,626 (43.6)^b^	676,170 (35.0)^b^
Missing	+	+	+	+	+	+	84 (0.0)	+	81 (0.0)

Abbreviations: —, none reported; +, small cell size of <20 observations.

a Data presented are number (%) unless otherwise indicated. Percentages may not add up to 100 due to rounding.

b Significant difference within state or county, between infants that died and those that lived (*P* ≤ .05).

c American Indian or Alaska Native, Asian Indian, Chinese, Filipino, Japanese, Korean, Vietnamese, other Asian, Native Hawaiian, Guamanian or Chamorro, Samoan, Other Pacific Islander, and other — not specified.

d Operationalized as inadequate prenatal care, defined as prenatal care that began after the fourth month of pregnancy and the mother had less than 50% of recommended prenatal care visits, versus other categories combined (intermediate to adequate plus, ie, prenatal care that began by the fourth month of pregnancy and the mother had 50% or more of recommended prenatal care visits).

e Aged <20 years, body mass index percentile ≥95th percentile; aged ≥20 years, body mass index ≥30, calculated as weight (in pounds) divided by height (in inches and squared) and the quotient multiplied by 703.

f Prepregnancy or pregnancy-induced. Hypertension included preexisting or gestational hypertension/preeclampsia or eclampsia. Diabetes included diagnosis before pregnancy or diagnosis during pregnancy.

After adjusting for variables included in the model, a few variables remained significantly associated with increased odds of infant death in both counties (Table 2). In Hidalgo County, mothers who had a high school education or less (aOR, 1.48; 95% CI, 1.20–1.90), had multiple gestation (aOR, 3.67; 95% CI, 2.57–5.23), or had cesarean delivery (aOR, 1.66; 95% CI, 1.33–2.06) had higher odds of infant death. Similarly, in Smith County, mothers who were unmarried (aOR, 1.65; 95% CI, 1.14–2.40), had multiple gestation (aOR, 3.11; 95% CI, 1.58–5.60), or had cesarean delivery (aOR, 1.78; 95% CI, 1.20–2.63) had higher odds of infant death. However, for Texas overall, several sociodemographic and pregnancy-related factors were significantly associated with infant death. Mothers who had a high school education or less, were unmarried, were non-Hispanic Black, had Medicaid or other/self-pay insurance, smoked cigarettes during pregnancy, had prepregnancy obesity, maternal hypertension, multiple gestation, or cesarean delivery were at increased odds of having an infant death. To see if there were any patterns to the missing data, sociodemographic characteristics of those with complete and missing data for the variables of interest for the overall Texas model were compared. Women with missing data in the overall model were more likely have a high school education or less, Hispanic, not married, non-US born, and with Medicaid insurance. In addition, sensitivity analysis using multiple imputation methods confirmed our findings for the overall model.

**Table 2:** Associations of Maternal Sociodemographic and Pregnancy Characteristics With Infant Deaths, Healthy Families Counties and Texas, 2011–2015^a^

Characteristic	Hidalgo County, aOR (95% CI) (N = 80,431)	Smith County, aOR (95% CI) (N = 15,173)	Texas, aOR (95% CI) (N = 1,744,178)
Maternal age, y	**—**	**—**	1.00 (1.00–1.01)
Education			
At least some college education	1 [Reference]	—	1 [Reference]
High school graduate or less	1.48 (1.20–1.90)^b^	—	1.39 (1.31–1.46)^b^
Marital status			
Married	—	1 [Reference]	1 [Reference]
Unmarried	—	1.65 (1.14–2.40)^b^	1.09 (1.03–1.15)^b^
Race and ethnicity			
Hispanic	—	—	1.03 (0.97–1.09)
Non-Hispanic Black	—	—	1.81 (1.69–1.94)^b^
Non-Hispanic White	—	—	1 [Reference]
Other^c^ or unknown	—	—	1.04 (0.93–1.17)
US-born mother			
Yes	—	—	1 [Reference]
No	—	—	0.82 (0.77–0.87)
Principal source of payment			
Private	—	—	1 [Reference]
Medicaid	—	—	1.13 (1.06–1.20)^b^
Other or self-pay	—	—	1.28 (1.18–1.38)^b^
Any cigarette smoking during pregnancy			
No	—	—	1 [Reference]
Yes	—	—	1.56 (1.42–1.70)^b^
Adequacy of Prenatal Care Utilization Index^d^			
Intermediate to adequate plus	—	—	1 [Reference]
Inadequate	—	—	0.97 (0.91–1.02)
Prepregnancy body mass index			
Overweight, normal, or underweight	1 [Reference]	1 [Reference]	1 [Reference]
Obese^e^	1.12 (0.90–1.41)	1.34 (0.90–1.98)	1.22 (1.16–1.28)^b^
Maternal diabetes^f^			
No	1 [Reference]	—	—
Yes	1.40 (0.98–2.01)	—	—
Maternal hypertension^f^			
No	—	—	1 [Reference]
Yes	—	—	1.11 (1.02–1.20)^b^
Multiple gestation			
No	1 [Reference]	1 [Reference]	1 [Reference]
Yes	3.67 (2.57–5.23)^b^	3.11 (1.58–5.60)^b^	4.04 (3.76–4.33)^b^
Mother transferred for maternal or fetal indications for this delivery			
No	1 [Reference]	1 [Reference]	1 [Reference]
Yes	14.48 (6.28–33.37)^b^	3.53 (0.85–9.71)	6.38 (5.40–7.52)^b^
Final delivery route			
Vaginal	1 [Reference]	1 [Reference]	1 [Reference]
Cesarean	1.66 (1.33–2.06)^b^	1.78 (1.20–2.63)^b^	1.29 (1.23–1.36)^b^

Abbreviation: aOR, adjusted odds ratio; —, not included in the model because they were not significant at the bivariate level.

a Those with missing information were excluded so numbers will not align with Table 1.

b *P* value ≤ .05.

c American Indian or Alaska Native, Asian Indian, Chinese, Filipino, Japanese, Korean, Vietnamese, other Asian, Native Hawaiian, Guamanian or Chamorro, Samoan, Other Pacific Islander, and other — not specified.

d Operationalized as inadequate prenatal care, defined as prenatal care that began after the fourth month of pregnancy and the mother had less than 50% of recommended prenatal care visits, versus other categories combined (intermediate to adequate plus, ie, prenatal care that began by the fourth month of pregnancy and the mother had 50% or more of recommended prenatal care visits).

e Aged <20 years, body mass index percentile ≥95th percentile; aged ≥20 years, body mass index ≥30, calculated as weight (in pounds) divided by height (in inches and squared) and the quotient multiplied by 703.

f Prepregnancy or pregnancy-induced. Hypertension included preexisting or gestational hypertension/preeclampsia or eclampsia. Diabetes included diagnosis before pregnancy or diagnosis during pregnancy.

### Causes of infant death

For infants born in 2015, the leading cause of infant death in both counties was congenital malformations, deformations, and chromosomal anomalies accounting for 39% and 26% of infant deaths in Hidalgo and Smith counties, respectively (Table 3). The other prevalent causes that were common to both counties were disorders related to short gestation and low birth weight, sudden infant death syndrome, and newborns affected by maternal complications of pregnancy. The 2015 ranking of leading causes for infant deaths for Hidalgo and Smith counties were similar to those for Texas overall, where the leading causes of infant death were congenital malformations, deformations, and chromosomal anomalies; disorders related to short gestation and low birthweight; sudden infant death syndrome; newborns affected by maternal complications of pregnancy; and accidents (unintentional injuries).

**Table 3:** Five Leading Causes of Infant Deaths, Healthy Families Sites, Infants Born in 2015

Cause of Death^a^ (ICD-10 Code)	Rank^b^	No. of deaths	Percentage of all infant deaths^c^
**Hidalgo County**			
All causes	—	80	100.0
Congenital malformations, deformations, and chromosomal abnormalities (Q00–Q99)^d^	1	31	39
Bacterial sepsis of newborn (P36)^d^	2	5	6
Disorders related to short gestation and low birthweight, not elsewhere classified (P07)^d^	3	4	5
Newborn affected by maternal complications of pregnancy (P01)^d^	4	3	4
Assault (*U01, X85–Y09)^d^	4	3	4
Diarrhea and gastroenteritis of infectious origin (A09)^d^	5	2	3
Sudden infant death syndrome (R95)^d^	5	2	3
All other causes^e^	—	30	38
**Smith County**			
All causes	—	23	100.0
Congenital malformations, deformations, and chromosomal abnormalities (Q00–Q99)^d^	1	6	26
Sudden infant death syndrome (R95)^d^	2	4	17
Newborn affected by maternal complications of pregnancy (P01)^d^	3	3	13
Disorders related to short gestation and low birthweight, not elsewhere classified (P07)^d^	3	3	13
Neonatal hemorrhage (P50–P52, P54)^d^	4	1	4
Diseases of the circulatory system (I00–I99)^d^	4	1	4
In situ neoplasms, benign neoplasms and neoplasms of uncertain or unknown behavior (D00–D48)^d^	4	1	4
All other causes^f^	—	4	17

Abbreviations: ICD-10, International Classification of Diseases, Tenth Revision; —, not applicable.

a An asterisk preceding a cause-of-death code indicates that the code is not included in ICD-10.

b Based on number of deaths.

c Percentages may not add up to 100 because of rounding.

d Causes labeled are ranked to determine leading causes of infant death.

e All other causes include all other causes (residual) (n = 21), neonatal hemorrhage (n = 1), respiratory distress (n = 1), accidents (n = 1), newborn affected by placental complications (n = 1), hydrops fetalis (n = 1), renal failure (n = 1), congenital pneumonia (n = 1), interstitial pneumonia (n = 1), and acute bronchitis (n = 1).

f All other causes include all other causes (residual) (n = 4).

### Zip code–level distribution of infant mortality rate and key maternal risk factors

**Figure 1:**
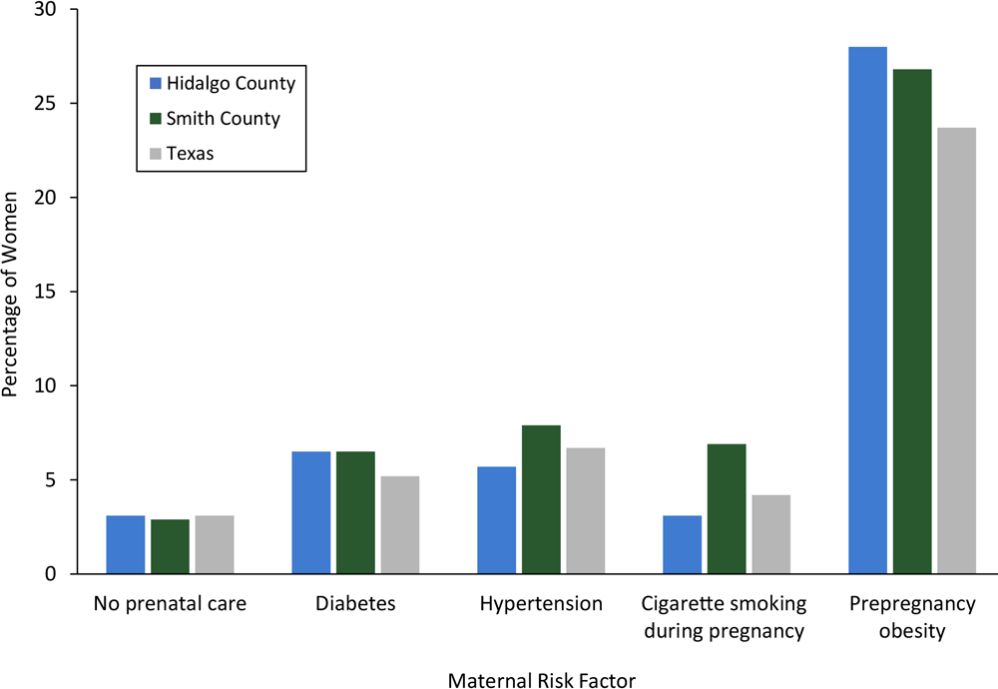
Percentage of women with key maternal risk factors, Healthy Families sites and Texas, 2011–2015. Hypertension included preexisting or gestational hypertension/preeclampsia or eclampsia; diabetes included diagnosis before pregnancy or diagnosis during pregnancy.

About 27% to 28% of women in the Healthy Families counties had prepregnancy obesity, whereas the state average was around 24% (Figure 1). Prevalence of no prenatal care, diabetes, and hypertension was 2.9% to 7.9% in the 2 counties, similar to state averages; however, the prevalence of maternal cigarette smoking during pregnancy in Smith County was 6.9%, which was higher than the prevalence in Hidalgo County (3.1%) and overall in the state (4.2%).

**Table:** 

Maternal Risk Factor	Hidalgo County	Smith County	Texas
No prenatal care	3.1	2.9	3.1
Diabetes	6.5	6.5	5.2
Hypertension	5.7	7.9	6.7
Cigarette smoking during pregnancy	3.1	6.9	4.2
Prepregnancy obesity	28.0	26.8	23.7

**Figure 2:**
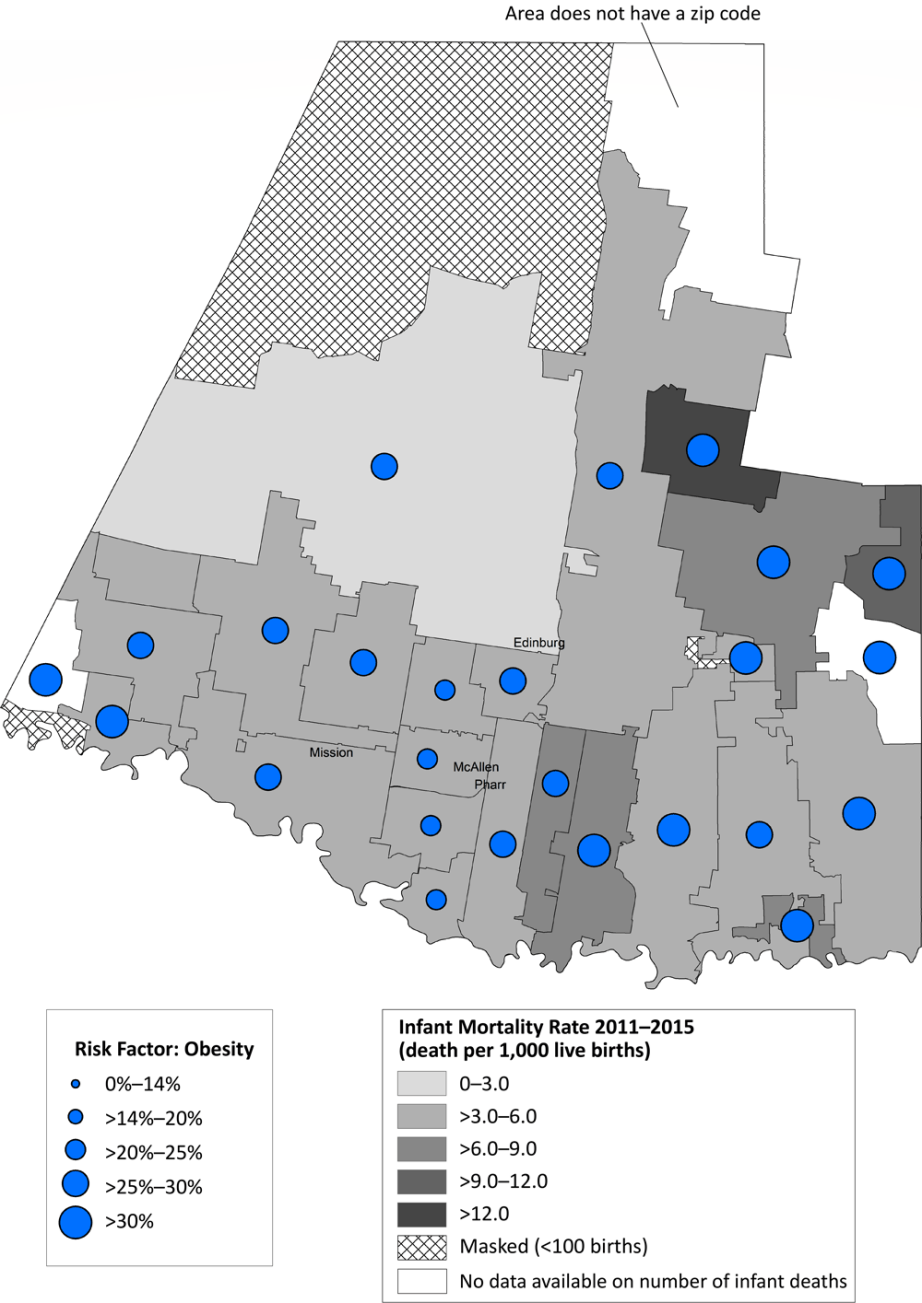
Infant mortality rate (deaths per 1,000 live births) with prevalence of prepregnancy obesity, by zip code area, Hidalgo County, Texas, 2011–2015.

**Figure 3:**
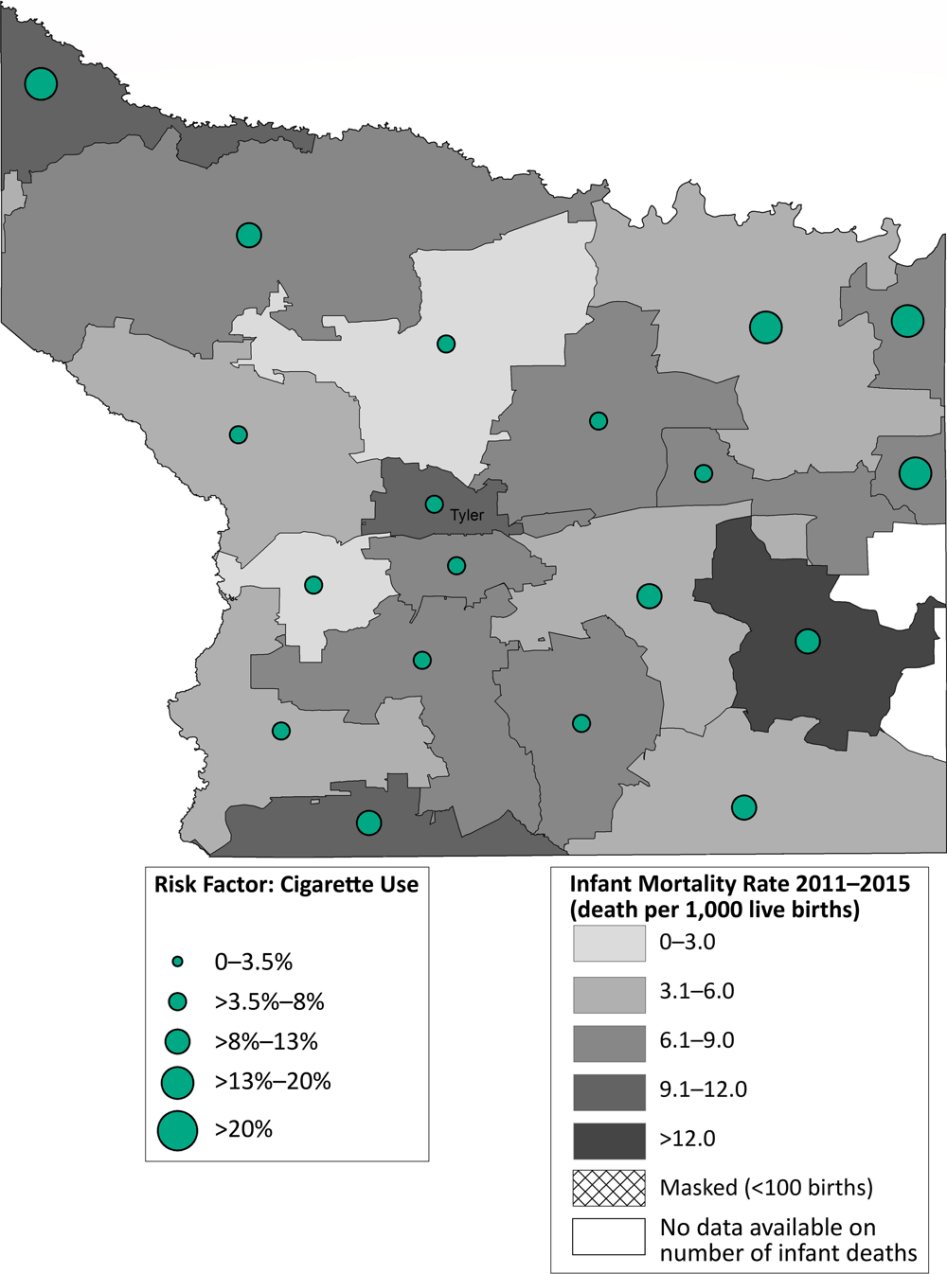
Infant mortality rate (deaths per 1,000 live births) with prevalence of cigarette smoking during pregnancy, by zip code area, Smith County, Texas, 2011–2015.

Most zip codes in Hidalgo County had an IMR below the state average of 5.4 per 1,000 live births (Figure 2). One zip code in the northeastern part of the county had an IMR greater than 12.0 per 1,000 live births. Hidalgo County had a high prevalence of prepregnancy obesity, particularly in those zip codes with high IMRs. Contrastingly, most zip codes in Smith County had an IMR higher than the state average (Figure 3). Most of these zip codes also had a high prevalence of maternal cigarette smoking during pregnancy.

## Discussion

In the Healthy Families initiative in 2 Texas counties with high prevalences of maternal chronic conditions, we observed that several maternal sociodemographic and pregnancy-related factors were associated with higher IMR. Additionally, wide variations in IMR and key maternal risk factors were observed at a more granular geographic level within the 2 counties. Maternal marital status, education, multiple gestation, and cesarean delivery were significantly associated with infant mortality. The leading cause of infant death in both counties for infants born in 2015 was congenital malformations, deformations, and chromosomal anomalies, which was similar to Texas and the national prevalence in 2016 (12,15).

In 2011 through 2015, the IMRs in Hidalgo County and Texas were below the Healthy People 2020 objective of 6.0 per 1,000 live births; however, the rate of infant mortality in Smith County was higher than the Healthy People 2020 objective (16). Individual-level risk factors associated with IMR in the 2 selected counties are supported by prior literature: education (15), being unmarried (17), multiple gestation (18), and cesarean delivery (19); and in Texas, being non-Hispanic Black (15), having Medicaid insurance or other/self-pay (20), and having maternal risk factors such as cigarette smoking during pregnancy, prepregnancy obesity, and hypertension (3). Another potential source of the lower IMR in Hidalgo County versus Smith County and the state overall is that the health of infants with non–US-born mothers may be better than infants with US-born mothers, which was consistent with our state model but not with our county-level models (21–23). Of note, because of low frequencies of infant mortality in the 2 selected counties, some risk factors that were significant for the Texas model were not significant for the individual county models.

Within the 2 counties, geographic variations existed at the zip code level. This was particularly true in Smith County, where a few zip codes had IMRs greater than 12 per 1,000 live births, double the Healthy People 2020 objective. Further, prevalence of key maternal risk factors such as prepregnancy obesity, diabetes, hypertension, and no prenatal care in the 2 counties were similar to the state average (3); however, when examined at a more granular level, several zip codes had high rates of prepregnancy obesity. Maternal cigarette smoking prevalence in Smith County was higher than the state average of 3.6% in 2015 (3), which has yet to reach the Healthy People 2020 objective of 1.4% maternal cigarette smoking during pregnancy (16). During the Healthy Families initiative, zip code–level analyses helped identify communities at an increased risk because of a high prevalence of infant mortality and key maternal risk factors, which resulted in increased focus on these regions. For example, in both counties, community health workers focused recruitment strategies to engage women from the most disproportionately affected zip codes. In Smith County, the Nurse–Family Partnership client base was adjusted to ensure women from communities at highest risk for infant mortality were being served. In Hidalgo County, the mobile health unit that provided contraception and pregnancy-related services was parked in communities with a high prevalence of key maternal risk factors (4). In addition, in Smith County, project partners and collaborators were made aware of the high prevalence of maternal cigarette smoking in certain zip codes; these results informed smoking cessation efforts in the county.

A key limitation of our study is that we did not account for social determinants of health, including structural racism that drives infant mortality, particularly among non-Hispanic Black infants (24), which may lead to some residual confounding. Another limitation is that we relied on vital records data, where medical risk factors such as diabetes, hypertension, and self-reported weight tend to be underreported compared with medical records (25–27), which may explain some of the null findings observed in the county-specific models (25). A third limitation is that we did not stratify our models by race and ethnicity because of low frequencies for infant deaths in the different groups. To reduce overadjustment bias, the models did not control for preterm birth, low birthweight, or gestational age because those are likely intermediates between maternal risk factors and infant death (28). Additionally, to maintain compliance with the data use agreement, the analyses were limited to zip code–level maps, because census tract–level analysis would result in many areas with less than 100 births over the study period. Our study had many strengths, including its large sample size, examination of several maternal factors with mutual adjustment in statistical models, and the geographic area–level analyses. Future studies should examine linking these data to more robust population health data to integrate relevant social determinants of health.

Data from this study were critical for driving strategies to better serve the health care needs of women residing in the 2 Healthy Families project sites, including focusing service delivery and outreach to maximize reach of services within disproportionately affected communities. Findings from this study were integrated into the planning, implementation, and monitoring of progress toward reducing infant mortality in the 2 counties and can inform broader efforts to improve pregnancy-related outcomes across the state.
